# Modeled estimates of HIV-serodifferent couples in tuberculosis-affected households in four sub-Saharan African countries

**DOI:** 10.1371/journal.pgph.0002609

**Published:** 2024-05-02

**Authors:** Meixin Zhang, Ashley S. Tseng, Godwin Anguzu, Ruanne V. Barnabas, J. Lucian Davis, Andrew Mujugira, Abraham D. Flaxman, Jennifer M. Ross

**Affiliations:** 1 Institute for Health Metrics and Evaluation, University of Washington, Seattle, Washington, United States of America; 2 Department of Epidemiology, University of Washington, Seattle, Washington, United States of America; 3 Department of Global Health, University of Washington, Seattle, Washington, United States of America; 4 PART Fellowship, Makerere University, Kampala, Uganda; 5 Department of Social Science Research Institute, Duke University, Durham, North Carolina, United States of America; 6 Division of Infectious Diseases, Massachusetts General Hospital, Boston, Massachusetts, United States of America; 7 Department of Medicine, Harvard Medical School, Boston, Massachusetts, United States of America; 8 Department of Epidemiology of Microbial Diseases, Yale School of Public Health, New Haven, Connecticut, United States of America; 9 Center for Methods in Implementation and Prevention Science, Yale School of Public Health, New Haven, Connecticut, United States of America; 10 Pulmonary, Critical Care, and Sleep Medicine Section, Yale School of Medicine, New Haven, Connecticut, United States of America; 11 Infectious Diseases Institute, Makerere University, Kampala, Uganda; 12 Department of Health Metrics Sciences, University of Washington, Seattle, Washington, United States of America; 13 Division of Allergy and Infectious Diseases, Department of Medicine, University of Washington, Seattle, Washington, United States of America; Texas Biomedical Research Institute, UNITED STATES

## Abstract

Household-based tuberculosis (TB) contact evaluation may be an efficient strategy to reach people who could benefit from oral pre-exposure prophylaxis (PrEP) because of the epidemiological links between HIV and TB. This study estimated the number of HIV serodifferent couples in TB-affected households and potential HIV acquisitions averted through their PrEP use in 4 TB-HIV high-burden countries. We conducted a model-based analysis set in Ethiopia, Kenya, South Africa, and Uganda using parameters from population-based household surveys, systematic literature review and meta-analyses, and estimates from the Global Burden of Diseases, Injuries, and Risk Factors Study 2019. We parameterized the nonlinear relationship between the proportion of serodifferent couples among people living with HIV and population-level HIV prevalence using Markov chain Monte Carlo methods. We integrated all parameters in a mathematical model and propagated uncertainty using a Monte Carlo approach. We estimated the HIV prevalence among adults aged 15–49 living in TB-affected households to be higher than in the general population in all 4 countries. The proportion of serodifferent couples among all couples in TB-affected households was also higher than in the general population (South Africa: 20.7% vs. 15.7%, Kenya: 15.7% vs. 5.7%, Uganda: 14.5% vs. 6.0%, Ethiopia: 4.1% vs. 0.8%). We estimated that up to 1,799 (95% UI: 1,256–2,341) HIV acquisitions in South Africa could be prevented annually by PrEP use in serodifferent couples in TB-affected households, 918 (95% UI: 409–1,450) in Kenya, 686 (95% UI: 505–871) in Uganda, and 408 (95% UI: 298–522) in Ethiopia. As couples in TB-affected households are more likely to be serodifferent than couples in the general population, offering PrEP during household TB contact evaluation may prevent a substantial number of HIV acquisitions.

## Introduction

Despite their different modes of transmission, human immunodeficiency virus (HIV) and tuberculosis (TB) are linked epidemics, with TB as the leading cause of death among persons living with HIV globally [1]. Ethiopia, Kenya, Uganda, and South Africa are all included on the World Health Organization’s (WHO) list of high-burden countries for HIV-associated TB, with an HIV-positive TB incidence in 2021 of 6.2, 60, 63, and 274 per 100,000 population, respectively [1]. Integration of HIV and TB care into “one-stop shop” models where patients can access care and prevention for HIV and TB concurrently has been central to global strategies to reduce TB and HIV morbidity and mortality for more than a decade [2].

Household TB contact (HHC) evaluation involves finding the HHCs of persons with TB, providing screening for active TB disease, and initiating TB preventive treatment (TPT) in persons without symptoms of active TB to reduce their risk of developing active TB disease [3, 4]. The United Nations (UN) High-Level Meeting on Tuberculosis in 2018 set a target to reach 24 million HHCs globally for TPT by 2022, which represented a commitment to a massive scale-up of HHC evaluation [5]. HIV prevalence among HHC is frequently higher than in the general population [6, 7]. HIV testing and linkage to care is recommended for HHCs [8] and implementation strategies are being tested in many high-burden settings [9, 10]. However, as HIV incidence may also be elevated among HHC [11], HHC programs may also offer a valuable opportunity to integrate HIV prevention counseling and pre-exposure prophylaxis (PrEP) initiation for individuals who test negative for HIV and could benefit from PrEP.

HIV-serodifferent couples (SDCs) involve relationships where one partner has HIV and the other does not [12]. SDCs are a priority population for PrEP under WHO [13], Ethiopia [14], Kenya [15], South Africa [16], and Uganda [17] PrEP guidelines because the HIV-negative partner is at elevated risk of acquiring HIV until their partner living with HIV links to antiretroviral therapy (ART) and achieves viral suppression. With the scale-up of HHC evaluation, there is an opportunity to increase the value of this service by integrating HIV screening to identify SDCs in TB-affected households (i.e., households in which at least one family member has been diagnosed with TB). Past studies have shown high PrEP uptake, adherence [18–22] and acceptability [18, 23] among SDCs, reporting that couple-focused ART and PrEP services were perceived to strengthen couples’ relationships [24] and could help support safer conception among couples desiring pregnancy [21, 22]. Thus, HHC evaluation may provide an excellent opportunity to improve linkage to HIV care and PrEP among SDCs and prevent HIV acquisition and adverse health effects. However, the number of SDCs in TB-affected households in high-burden settings has not yet been measured or estimated.

Mathematical models that integrate multiple sources of health surveillance data can be used to compare the estimated numbers of individuals who would benefit from PrEP using different screening strategies. Estimates of the potential opportunity for HIV screening and testing embedded within HHC programs, a natural next step in HIV-TB care integration, are lacking in high HIV burden settings. In this study, we compare the efficiency of identifying SDCs through TB HHC investigation versus population screening using modeled scenarios in four TB-HIV high-burden countries (Ethiopia, Kenya, South Africa, and Uganda) and estimate the potential HIV acquisitions averted by PrEP.

## Methods

### Study design

This model-based analysis incorporates modeled estimates with parameters from the literature to generate estimates of the prevalence of SDCs among TB-affected households in four high-burden countries (Ethiopia, Kenya, South Africa, and Uganda). These four countries were selected for several reasons. First, they were identified as high HIV-associated TB burden countries by the WHO [1]. Second, while they all have high prevalence of HIV-associated TB, they represent a relatively wide range of HIV prevalence in the general population, from 0.9% of people ages 15–49 years old in Ethiopia [25] living with HIV in 2016, to 4.5% in Kenya [26], 6.0% in Uganda [27], and 21.2% in South Africa [28]. Through our model-based analysis, we explored how the HIV and TB epidemics intersect in different settings. Lastly, each of these countries had the necessary data inputs for our model. We limited our study cohort to adults aged 15–49 years due to elevated HIV prevalence within this age group and data availability from national surveys. As HIV prevalence among people with incident TB tends to be higher than among people with prevalent TB [29], we performed two sets of estimation using parameters corresponding to incidence versus prevalence scenarios.

This analysis complies with the Guidelines for Accurate and Transparent Health Estimates Reporting (GATHER) statement [30]. It uses publicly-available, non-identifiable data and did not require human subjects review. We conducted the analysis in Stata/SE 15.1 and R (version 4.0.5).

### Data sources and parameter development

The data sources for our analysis include the Global Burden of Diseases, Injuries, and Risk Factors 2019 Study (GBD 2019) [31], population-based surveys, and literature (**Table 1**). We used GBD 2019 estimates of the number of people with prevalent TB and the number of people with incident TB by age, sex, and country and the number of people with incident TB who were also living with HIV [31]. We used a GBD-based analysis for the household composition of people with TB [32]. We calculated the number of people living with HIV among people with prevalent TB from national TB prevalence surveys (**S1 Text**). We conducted analyses to generate key model parameters that are detailed below, including (1) country-specific ratios comparing HIV prevalence among household members of people with TB to HIV prevalence in the general population and (2) a general relationship comparing the proportion of SDC among all people living with HIV who are in stable partnerships (*P*_*SDC*_) to population-level HIV prevalence.

**Table 1:** Data sources for prevalence and incidence modeling scenarios.

Parameter	Ethiopia	South Africa	Kenya	Uganda
**National HIV prevalence** [25–28]				
Male				
15–24	0.1%	3.4%	0.6%	0.8%
25–34	0.4%	17.7%	2.7%	4.5%
35–44	1.1%	26.6%	5.3%	9.9%
45–49	1.6%	19.4%	8.3%	14.0%
Female				
15–24	0.3%	11.6%	2.3%	3.4%
25–34	1.5%	36.0%	7.3%	9.8%
35–44	2.5%	40.3%	10.3%	12.5%
45–49	1.9%	19.9%	10.6%	12.8%
**Proportion of people with prevalent TB also living with HIV** [33–35]*				
Male				
15–24	-	-	7.04%	6.94%
25–34	-	-	14.90%	45.01%
35–44	-	-	22.07%	26.98%
45–49	-	-	9.46%	32.38%
Female				
15–24	-	-	12.87%	7.81%
25–34	-	-	27.26%	50.68%
35–44	-	-	40.36%	30.38%
45–49	-	-	17.30%	36.45%
Both sexes, all ages	-	28.80%	-	-
**HIV prevalence among people with notified TB** [36]				
	5.38%	-	-	-
**Ratio of HIV prevalence among people with prevalent TB versus people with notified TB** [29]				
	0.47(0.34–0.65)	-	-	-
**Number of people with incident TB (Incidence scenario)** [31]**±**				
Male				
15–24	31,056	28,675	17,632	16,501
25–34	27,577	49,240	17,418	13,247
35–44	19,406	40,945	16,812	9,939
45–49	7,716	12,781	6,968	4,020
Female				
15–24	31,751	62,720	18,459	16,926
25–34	26,863	79,842	21,604	13,018
35–44	18,441	41,518	16,552	6,767
45–49	5,759	10,698	4,974	2,155
**Number of people with prevalent TB (Prevalence scenario)** [31] **±**				
Male				
15–24	46,052	37,773	28,957	27,062
25–34	40,505	63,935	28,285	21,235
35–44	28,155	58,233	25,634	15,176
45–49	10,684	17,059	8,782	5,331
Female				
15–24	48,294	95,546	30,480	24,681
25–34	44,292	142,524	39,402	20,936
35–44	29,803	95,546	27,696	10,489
45–49	8,487	17,976	6,950	2,772
**Number of TB household contacts (Incidence scenario)** [32] **±**				
Male				
15–24	164,079	110,762	95,802	77,417
25–34	75,150	94,114	43,674	33,495
35–44	55,028	70,959	35,791	24,336
45–49	22,589	28,169	15,772	10,436
Female				
15–24	141,428	104,266	87,449	74,183
25–34	83,411	96,356	49,205	44,264
35–44	67,269	78,867	44,468	32,486
45–49	21,677	34,923	15,825	11,553
**Number of TB household contacts (Prevalence scenario)** [32] **±**				
Male				
15–24	258,094	172,370	156,188	121,932
25–34	119,249	149,299	73,313	53,452
35–44	88,584	113,859	60,240	38,777
45–49	35,999	44,041	25,910	16,559
Female				
15–24	222,049	162,374	143,169	117,426
25–34	132,615	145,784	82,261	70,966
35–44	105,672	118,245	72,253	51,367
45–49	33,929	52,733	25,528	18,107
**Proportion of adults engaged in stable partnerships** [25, 28, 37, 38]				
Male				
15–24	16.4%	3.5%	8.4%	15.4%
25–34	72.4%	36.1%	66.8%	79.0%
35–44	93.4%	57.7%	87.8%	89.5%
45–49	95.3%	63.6%	90.8%	89.3%
Female				
15–24	37.4%	51.4%	33.1%	40.7%
25–34	84.0%	43.3%	76.6%	77.8%
35–44	83.1%	53.7%	74.9%	75.5%
45–49	78.5%	51.0%	71.6%	69.6%

*Reporting of this parameter varied between the data sources for different countries. Calculating this parameter for Ethiopia and South Africa involved multiple data sources, as described in S1 Text. **±**These parameters each represent annual totals.

For the first key model parameter, we conducted a systematic review in PubMed for studies published from the four included countries that reported HIV prevalence among household members of people with TB. Further details about the systematic review are in **S1 Text**. We contacted authors to request age-stratified data from studies that did not report HIV prevalence data by age group. Then, we used random-effects meta-analysis to generate estimates of HIV prevalence among adult HHCs for each country. We extracted HIV prevalence in the general population from population-based surveys (**Table 1**) [25–28]. Lastly, we calculated the HIV prevalence ratio for each country as the HIV prevalence in adult HHCs (from the meta-analysis) divided by the HIV prevalence in the general adult population.

For the second key parameter, we completed several steps to model the relationship between the proportion of SDCs among all people living with HIV who are in stable partnerships (*P*_*SDC*_) to population-level HIV prevalence. We began by calculating *P*_*SDC*_, which is used to separate SDCs from all partnerships involving people living with HIV. We extracted data for HIV prevalence, prevalence of serodifference and seroconcordancy from 19 Demographic and Health Surveys (DHS) conducted between 2006 and 2018 in sub-Saharan Africa that incorporated serum HIV testing and calculated *P*_*SDC*_ from each of the included DHS (**S1 Text**). We then described the relationship between *P*_*SDC*_ and national HIV prevalence with a nonlinear one-parameter model using grid search (**S1 Text**), and fitted Markov chain Monte Carlo (MCMC) models to generate the uncertainty intervals. The relationship was described using the following equation:

$$P_{SDC}(P)≅\frac{{\left(P(1-P)\right)}^{\alpha }}{P}$$

where α is the homogeneity parameter in this nonlinear one-parameter model and *P* is national HIV prevalence among 15-49-year-olds. We aimed to find the α that describes this relationship most accurately. Using this relationship and the HIV prevalence in TB affected households that we estimated, we back-calculated *P*_*SDC*_ in TB affected households in Ethiopia, Kenya, Uganda, and South Africa for use in the estimation steps described below.

### Estimation model

**Fig 1:**
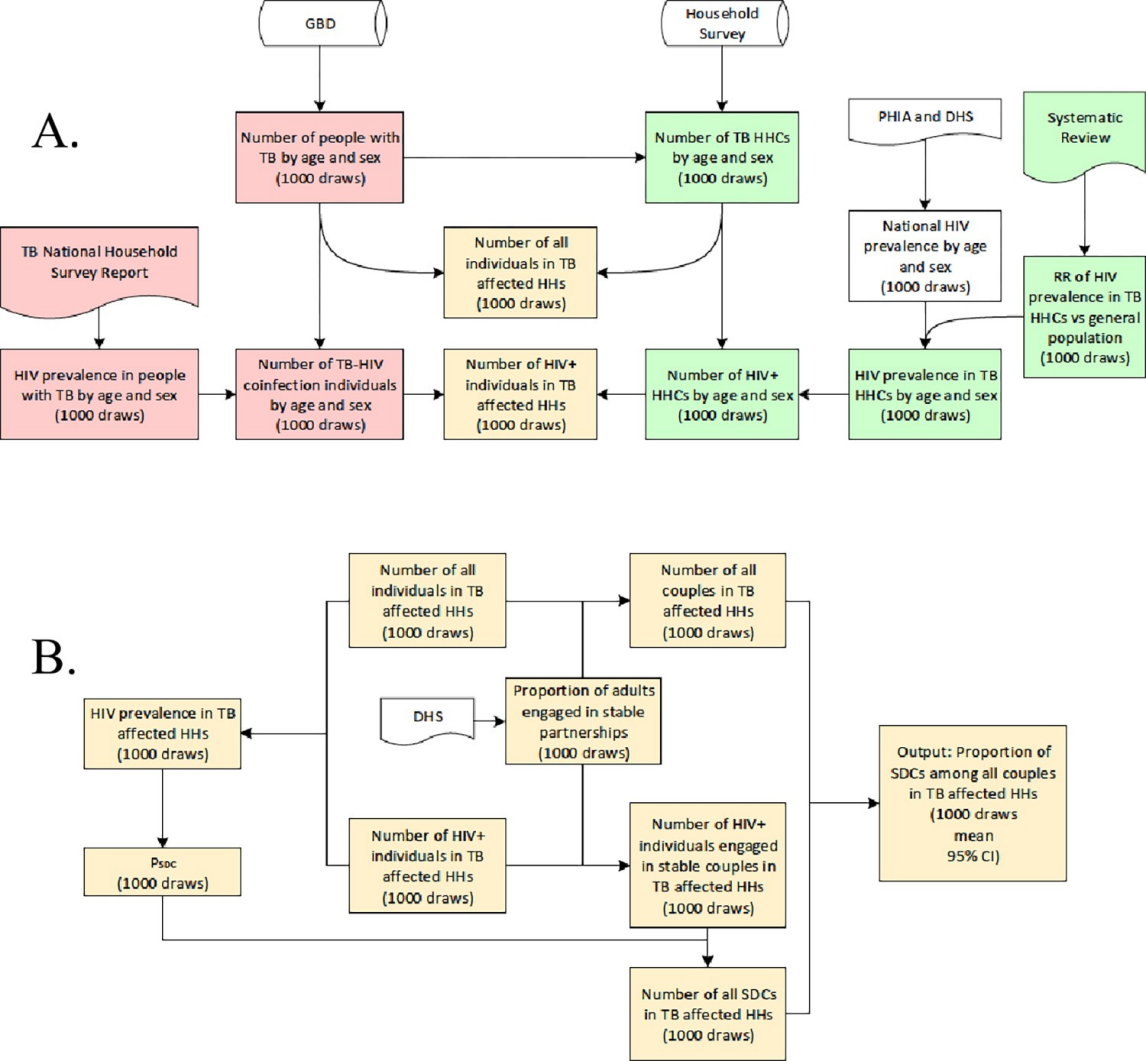
Flow diagrams of mathematical models used in this study. (A) Flow-chart depicting the process of estimating the number of individuals living in households affected by tuberculosis (TB) and the frequency of individuals living with human immunodeficiency virus (HIV) in these households. (B) Flow-chart depicting the process of generating the proportion of HIV-serodifferent couples (SDCs) using outputs from (A). Global Burden of Disease, Injuries, and Risk Factors Study (GBD); Demographic and Health Surveys (DHS); Households (HHs); Household contacts (HHC); Population-based HIV Impact Assessment (PHIA); the proportion of serodifferent couples among people living with HIV who are in stable partnerships (P_SDC_); Relative risk (RR); Serodifferent couples (SDCs).

The steps of the estimation model come together to calculate the proportion of SDCs among couples in TB-affected households. The model is described in **Fig 1**. The steps shown in **Fig 1A** were divided into two sections: the steps shown in pink were used to calculate the number of people with TB and with TB and HIV; the steps shown in green were used to calculate the number of HHC and the number of HHC living with HIV. By adding these two sections, we estimated the number of all individuals aged from 15 to 49 years old in TB affected households and the number of individuals living with HIV among them. Using these two estimates, we calculated the HIV prevalence in TB affected households (**Fig 1B**).

We used several steps to incorporate data on couples. First, we obtained the proportion of adults engaged in stable partnerships from DHS reports in each of the four countries (**Table 1**). Then, we applied this proportion to the number of adults in TB-affected households and divided the product by 2 to get the number of couples in TB-affected households. Similarly, we applied the proportion of adults engaged in stable partnerships to the number of individuals living with HIV to get the number of individuals living with HIV in TB-affected households who were engaged in stable partnerships. These partnerships include SDCs and HIV-positive seroconcordant partnerships. We multiplied *P*_*SDC*_ with number of individuals living with HIV in TB-affected households and engaged in stable partnerships to get the number of SDCs. We divided the number of SDCs by the number of all couples in TB-affected households to obtain the proportion of SDCs in TB-affected households.

In the final modeling step, we estimated the number of HIV acquisitions that could be averted through PrEP use among SDCs in TB-affected households. As HIV is not transmitted from individuals with a suppressed viral load, we needed to estimate the proportion of people living with HIV in TB-affected households without viral suppression, but we did not find studies to estimate the values of this parameter (search in **S1 Text**). Instead, we conducted a scenario analysis where 25%, 50%, 75%, and 100% of partnerships would be eligible for PrEP because the partner living with HIV was not virally suppressed. We used PrEP parameters from a PrEP demonstration study conducted among >1,000 SDCs in Kenya and Uganda [39]. From this study, PrEP uptake among HIV-negative participants was 97%, PrEP effectiveness against HIV transmission among SDCs was 95%, and the estimated HIV incidence among HIV-negative partners in SDCs who did not initiate PrEP was 4.75 per 100 person-years. With this information, we calculated the HIV infections in SDCs over 1 year when HIV-negative partners had not initiated PrEP using equation:

$$n*\frac{4.75}{100\;person-years}$$

Therefore, when 100% of partnerships were eligible for PrEP, the number of HIV infections among HIV-negative partners in 1 year is:

$$\left\{n*97\%*\frac{4.75}{100\;person-years}*(1‐95\%)\}+\{n*(1‐97\%)*\frac{4.75}{100\;person-years}\right\}$$

To estimate averted transmission from PrEP, we subtracted the HIV acquisitions when partnerships were eligible for PrEP from HIV acquisitions when PrEP was absent.

We used a Monte Carlo approach to propagate uncertainty levels throughout the model. For each measure, we generated 1,000 draws based on its mean estimate and uncertainty level or sample size and cases, with the assumption that data are normally distributed or log-spaced normally distributed. These 1,000 draws were randomly distributed, meaning they were not increasingly or decreasingly sorted. When we performed arithmetic operations on two measures with 1,000 draws, we did it on each draw, resulting in 1,000 draws of the output. The lower and the upper bounds of the 95% uncertainty level are at 2.5 and 97.5 percentiles of the 1,000 draws.

## Results

**Fig 2:**
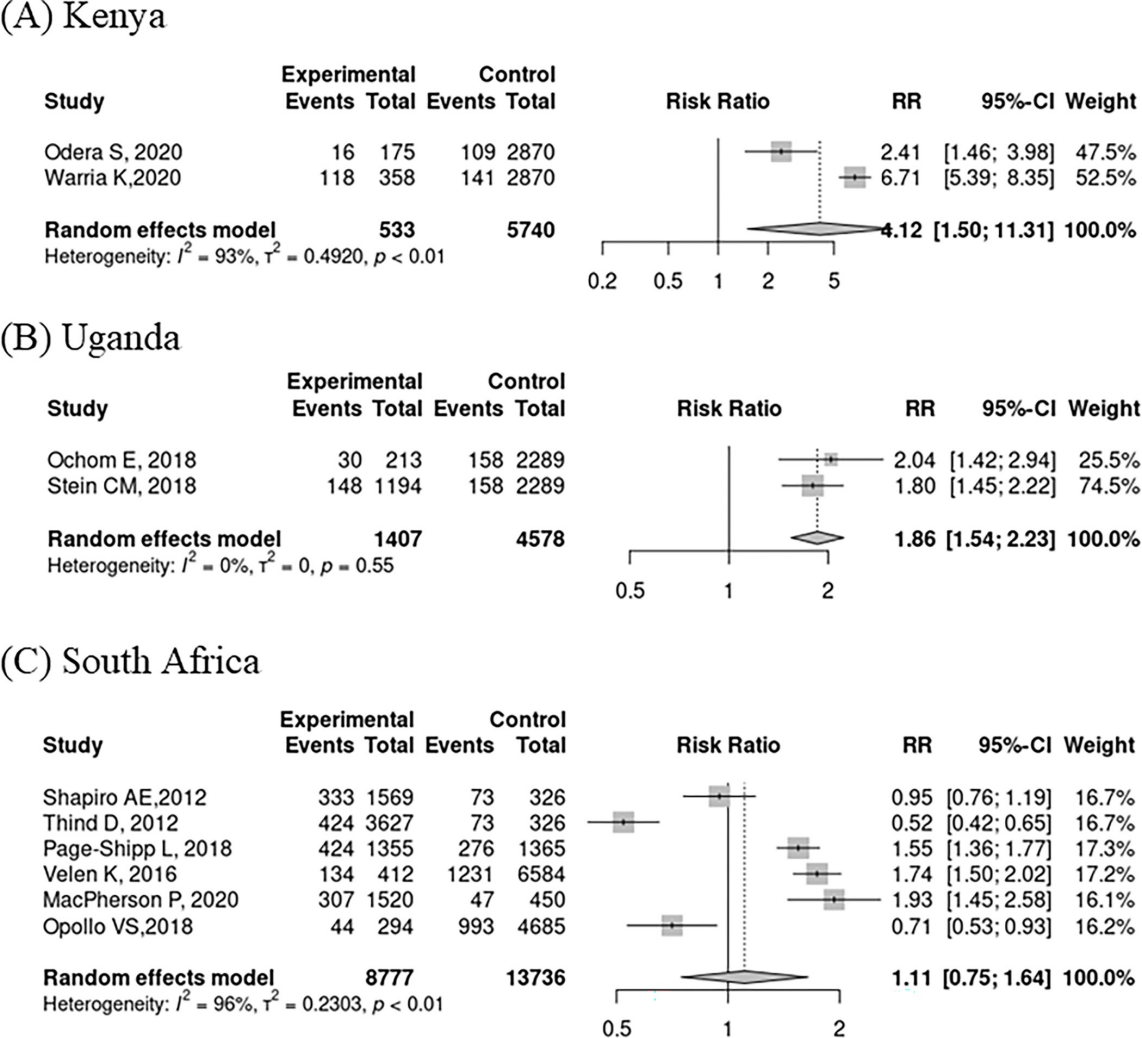
Forest plot of the relationship between HIV prevalence among adult household TB contacts (HHCs) vs. HIV prevalence in the general adult population in studies conducted in Kenya (A), Uganda (B), and South Africa (C).

The literature search for the HIV prevalence among adult household members of people with TB resulted in 502 PubMed records (**Fig A in S1 Text**). All 502 records proceeded to title/abstract screening, from which 19 reports were sought for full-text retrieval. After assessing 19 full text articles for eligibility, 9 were excluded. Full-text evaluation resulted in 10 studies that met all inclusion criteria [6, 7, 9, 40–46]. Two publications were from Kenya, 6 from South Africa, and 2 from Uganda. All were published between 2013–2021. We made forest plots by country using the HIV prevalence ratio among adult HHCs versus HIV prevalence in the general adult population (**Fig 2**). In Kenya (prevalence ratio [PR] = 4.12; 95% uncertainty interval [UI]: 1.50, 11.31) and Uganda (PR = 1.86; 95% UI: 1.54, 2.23), the HIV prevalence in HHCs was higher than the HIV prevalence in the general population. In South Africa, the HIV prevalence was similar to the general population (PR = 1.11; 95% UI: 0.75, 1.64). In Kenya, the heterogeneity of study estimates led to a wide UI.

**Fig 3:**
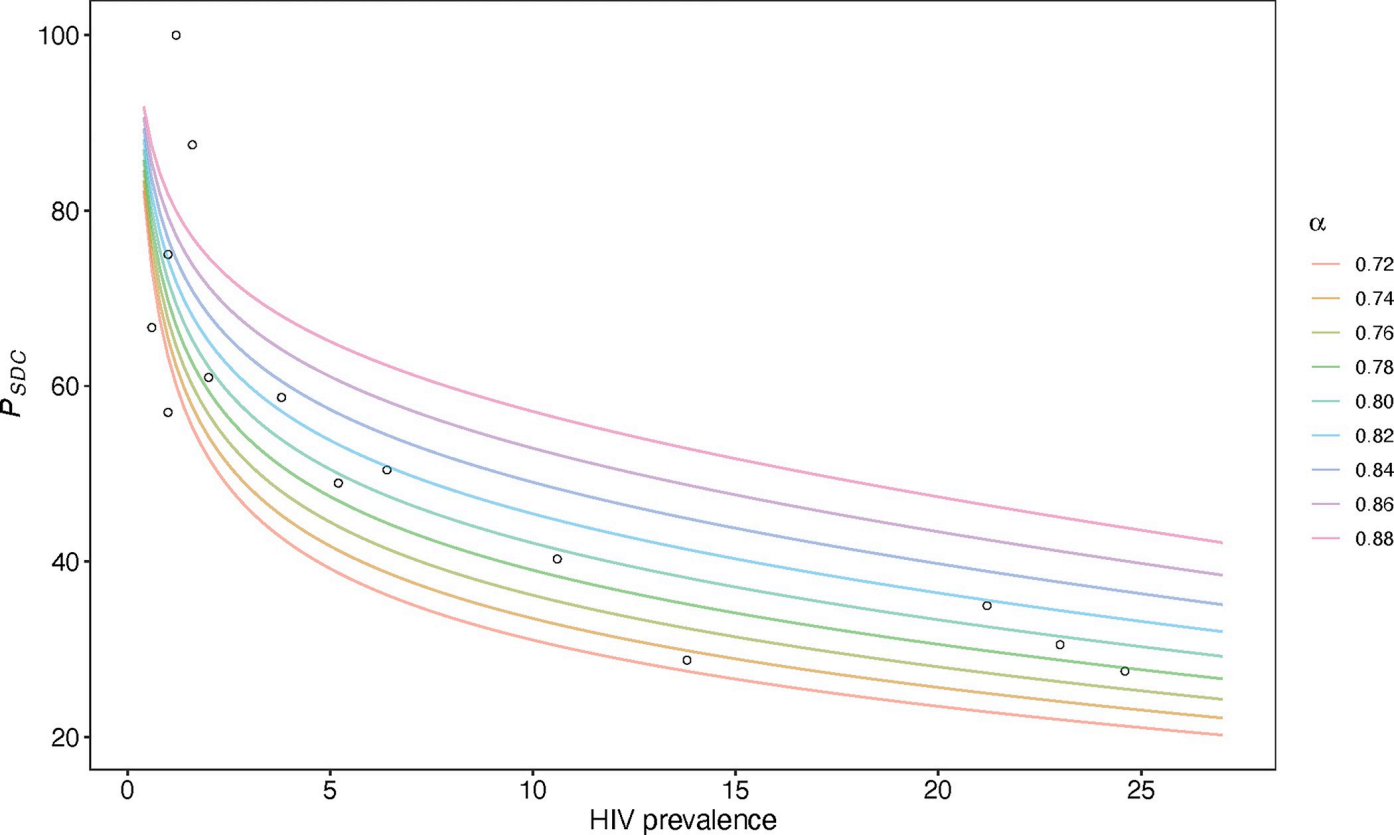
Simulated relationship between the proportion of SDC among all people living with HIV who are in stable partnerships (*P*_*SDC*_) and HIV prevalence using α between 0.7 and 0.9.

To describe the relationship between *P*_*SDC*_ and HIV prevalence (*P*), we found the best homogeneity parameter, α. As shown in **Fig 3**, each data point represents 1 *P*_*SDC*_−*P* pair extracted from DHS reports. We tested 9 values of α from 0.1 to 0.9 at 0.1 intervals and calculated the least square from the real *P*_*SDC*_ to predicted *P*_*SDC*_ to narrow our search window for the parameter value that minimized the least square. In this step, α = 0.8 minimized the least square. We further tested another 9 values of α from 0.72 to 0.88 and found that α = 0.82 minimized the least square (**Fig 3**). After we fitted α = 0.82 into MCMC, we found the estimated α is 0.829 (95% UI: 0.824–0.834).

### Estimated number and proportion of serodifferent couples

In the model-based analysis, we estimated the HIV prevalence among adults living in a household with someone with incident TB to be higher than in the general population in all 4 countries (South Africa: 33.0% vs. 20.6% [95% UI: 19.9%-21.3%], Kenya 19.9% vs. 4.5% [95% UI: 4.1%-4.9%], Uganda: 16.9% vs. 6.0% [95% UI: 5.7%-6.3%], Ethiopia: 3.1% vs. 0.9% [95% UI: 0.8%-1.0%]) (**Table 2**). The number of SDCs in households with incident TB ranged from 9,327 (95% UI: 7,988–10,790) in Ethiopia to 41,099 (95% UI: 33,957–48,299) in South Africa. The proportion of couples that were serodifferent was higher in TB-affected households than in the general adult population in all four countries (South Africa: 20.7% vs. 15.7%, Kenya: 15.7% vs. 5.7%, Uganda: 14.5% vs. 6.0%, Ethiopia: 4.1% vs. 0.8%) (Table 2). Couples in TB-affected households were approximately five times more likely to be serodifferent than couples in the general population in the country with the lowest HIV prevalence (Ethiopia) versus 2–3 times more likely in the countries with mid-range HIV prevalence (Uganda and Kenya), and <1.5 times in the country with the highest HIV prevalence (South Africa).

**Table 2:** The HIV prevalence and the SDCs in TB affected households (incidence modeling scenario).

Country	n of HIV+ in TB affected HHs	n of 15–49 aged people in TB affected HHs	HIV prevalence among TB affected HHs^i^	n of SDC in TB affected HHs^ii^	P of SDC in TB affected HHsiii	P of SDC in population^iv^
Kenya	100,963	508,403	19.85	21,004	15.73	5.7
	(31,253–187,150)	(429,876–597,960)	(6.2–35.52)	(9,482–31,279)	(7.14–22.27)	(4.4–7.0)
Uganda	66,000	390,743	16.90	15,683	14.53	6.0
	(56,703–77,107)	(340,703–442,779)	(15.11–18.81)	(13,651–17,862)	(13.49–15.63)	
Ethiopia	24,595	799,198	3.08	9,327	4.1	0.8
	(21,094–28,535)	(683,698–925,745)	(2.98–3.17)	(7,988–10,790)	(3.94–4.26)	(0.6–1.0)
South Africa	311,163	944,835	32.96	41,099	20.68	15.7
	(228,375–393,711)	(810,310–1,087,272)	(25.04–40.25)	(33,957–48,299)	(18.08–22.59)	(12.2–19.2)

i n of HIV+ in TB affected HHs / the number of 15–49 aged people in stable partnerships

^ii^ n of HIV+ in TB affected HHs * *P*_*SDC*_

^iii^ n of SDC / (n of 15–49 aged people in TB affected HHs / 2)

iv n of SDC / (n of 15–49 aged people in stable partnerships / 2)

### Serodifferent couples in households with prevalent TB

We estimated the same measures in a prevalence scenario, which was calculated among households of people with prevalent TB. The estimated HIV prevalence among adults in TB- affected households was similar to the estimates in the incidence scenario and remained higher than in the general population in all the 4 countries (**Table 3**). The number of SDCs among TB-affected households ranged from 6,744 (95% UI: 5,440–8,244) in Ethiopia to 60,038 (95% UI: 47,043–71,865) in South Africa. The proportion of SDC in TB-affected households was also higher than that in the general population (South Africa: 19.4% vs. 15.7%, Kenya: 14.9% vs. 5.7%, Uganda: 13.0% vs. 6.0%, Ethiopia: 1.9% vs. 0.8%).

**Table 3:** The HIV prevalence and the SDCs in TB affected households (prevalence modeling scenario).

Country	n of HIV+ in TB affected HHs	n of 15–49 aged people in TB affected HHs	HIV prevalence among TB affected HHs^i^	n of SDC in TB affected HHs^ii^	P of SDC in TB affected HHs^iii^	P of SDC in population^iv^
Kenya	154,026	835,048	18.45	32,775	14.93	5.7
	(39,011–296,038)	(704,750–988,343)	(4.64–34.15)	(12,317–50,639)	(5.71–21.95)	(4.4–7.0)
Uganda	88,312	616,268	14.34	22,137	13.00	6.0
	(72,481–105,520)	(534,913–701,832)	(12.26–16.44)	(18,631–25,723)	(11.70–14.21)	
Ethiopia	14,901	1,252,461	1.19	6,744	1.89	0.8
	(11,823–18,593)	(1,078,580–1,456,274)	(0.98–1.43)	(5,440–8,244)	(1.62–2.19)	(0.6–1.0)
South Africa	424,211	1,465,888	28.95	60,038	19.38	15.7
	(288,698–544,733)	(1,255,006–1,689,932)	(20.4–36.25)	(47,043–71,865)	(16.05–21.62)	(12.2–19.2)

^i^ n of HIV+ in TB affected HHs / the number of 15–49 aged people in stable partnerships

^ii^ n of HIV+ in TB affected HHs *P_*SDC*_

^iii^ n of SDC / (n of 15–49 aged people in TB affected HHs / 2)

^iv^ n of SDC / (n of 15–49 aged people in stable partnerships / 2)

### HIV acquisitions averted through PrEP among serodifferent couples

The estimated number of individuals prevented from acquiring HIV differed depending on the proportion of seronegative partners that were eligible for PrEP. If 100% of partners were eligible for PrEP, the HIV acquisitions averted through PrEP were estimated to range from 408 (95% UI: 298–522) in Ethiopia to 1,799 (95% UI: 1,256–2,341) in South Africa in the incidence scenario (**Table 4**). In 2019, these represented 0.56% of the annual HIV acquisitions estimated in GBD 2019 among people ages 15–49 in South Africa, 1.25% in Uganda, 1.62% in Kenya, and 2.21% in Ethiopia. In the prevalence scenario, the estimated number of HIV acquisitions averted ranged from 295 (95% UI: 206–396) in Ethiopia to 2,628 (95% UI: 1,800–3,457) in South Africa (**Table 5**). If most partners living with HIV were stable on ART and virally suppressed, then a smaller proportion of seronegative partners might initiate PrEP. For example, if 25% of partners were eligible for PrEP, the HIV acquisitions averted through PrEP was estimated to range from 102 (95% UI: 74–131) in Ethiopia to 450 (95% UI: 314–585) in South Africa in the incidence scenario (**Table 4**). In the prevalence scenario, it ranged from 74 (95% UI: 51–99) in Ethiopia to 657 (95% UI: 450–864) in South Africa (**Table 5**).

**Table 4:** HIV acquisitions averted through pre-exposure prophylaxis (PrEP) (incidence modeling scenario).

	Percentage of partnerships would be eligible for PrEP because the partner living with HIV was not virally suppressed			
	100%	75%	50%	25%
Kenya	918 (409–1,450)	689 (307–1,088)	459 (204–725)	230 (102–363)
Uganda	686 (505–871)	515 (379–654)	343 (253–436)	172 (126–218)
Ethiopia	408 (298–522)	306 (223–392)	204 149–261)	102 (74–131)
South Africa	1,799 (1,256–2,341)	1,349 (942–1,756)	900 (628–1,170)	450 (314–585)

**Table 5:** HIV acquisitions averted through PrEP (prevalence modeling scenario).

	Percentage of partnerships would be eligible for PrEP because the partner living with HIV was not virally suppressed			
	100%	75%	50%	25%
Kenya	1,433 (535–2,322)	1,075 (401–1,742)	716 (268–1,161)	358 (134–581)
Uganda	969 (696–1,240)	727 (522–930)	484 (348–620)	242 (174–310)
Ethiopia	295 (206–396)	221 (154–297)	148 (103–198)	74 (51–99)
South Africa	2,628 (1,800–3,457)	1,971 (1,350–2,593)	1,314 (900–1,728)	657 (450–864)

## Discussion

We conducted a model-based analysis to estimate the number of people in HIV serodifferent couples, a high-priority population for PrEP, that could be reached through HHC investigation in Ethiopia, Kenya, South Africa, and Uganda. We estimated that households affected by TB in all four countries were more likely to include SDC than households in the general population, indicating that HHC investigation may be an efficient strategy to find SDC for HIV testing and PrEP while partners with HIV initiate ART and become virally suppressed. However, the estimated number of HIV acquisitions prevented was a relatively small portion of those estimated to occur in each country annually.

We explored how HIV and TB intersect differently within households in different epidemiological contexts by conducting the analysis in countries representing a range of HIV and TB prevalence, despite all four countries being classified as having a high burden of HIV- associated TB by the WHO [1]. The HIV prevalence ratio for adult household contacts of people with TB compared to adults in the general population was higher in Kenya and Uganda than in South Africa, where adult HIV prevalence is three-fold higher than in Kenya and Uganda. While our meta-analysis identified heterogeneity between studies from South Africa, this finding aligns with the only study in our search that directly compared these values and found a slightly lower HIV prevalence among HHCs in North West Province, South Africa compared to people in randomly-selected control households in the same community [42]. A possible explanation for this finding is that HIV may be more generalized in South Africa where HIV prevalence is higher. Another difference observed for South Africa was that the proportion of people aged 15–49 years who are engaged in stable partnerships was lower than in the other countries, which impacted the total number of couples as well as the number of couples where one or more partner was living with HIV.

An intervention to offer PrEP to seronegative partners would be most impactful at preventing HIV acquisitions if partners living with HIV were not already engaged in care and virally suppressed. We found little published data about engagement in HIV care among SDC in TB-affected households, and so we explored this critical factor in scenario analysis. We assume that the partner with HIV will also have TB in the majority of SDC in TB-affected households, as was demonstrated in an analysis in Kampala, Uganda [47]. Several studies have found a high yield of new diagnoses of HIV among adult household TB contacts, including 6.3% in rural South Africa [48], while another study in urban Uganda found a relatively lower yield (2.3%) [9].

These findings have potential implications for health programs, particularly in the context of the commitment at the UN High-Level meeting for TB in 2018 to massively scale up HHC investigation to reach global targets for TPT initiation. While international guidelines recommend HIV testing as part of HHC investigation in high-prevalence settings [8], our findings suggest that it is also valuable to consider PrEP screening, particularly for partners of people with newly diagnosed TB. To that end, understanding acceptable and feasible approaches to PrEP screening and initiation or referral in the context of HHC investigation is an important goal that can build from existing knowledge of acceptable approaches to HIV testing in the context of HHC investigation [49], experience with PrEP screening and delivery in other non-clinical settings [50], and delivery strategies for ART and PrEP among SDC that include counseling to support couples [24].

Strengths of this study include the use of systematic literature reviews to develop model parameters and use of a modeling approach that propagates uncertainty throughout the estimation process. Additionally, this study generated a new analysis of the relationship between the proportion of SDC among all people living with HIV who are in stable partnerships and population-level HIV prevalence that builds from prior studies [51, 52].

The study also has limitations. Notably, we did not estimate the number of people in TB-affected households with substantial HIV exposures who were not in serodifferent relationships, which could provide a higher estimate of the number of people in TB-affected households who could benefit from PrEP [13]. In settings with available data, future work could include people with additional exposures. We also assumed that couples had monogamous relationships that were stable over time. Additionally, our meta-analysis did not account for subnational variation in HIV prevalence when comparing study estimates to HIV prevalence estimates in the general population due to the complexity of identifying an appropriate comparison estimate for each study location. Finally, we relied on some assumptions that are part of the other modeled estimates (e.g., that the household composition of households of people with TB is the same as for households without TB) [32].

## Conclusions

In summary, we estimated that households affected by TB in four HIV-TB high-burden countries were more likely to include HIV serodifferent couples than households in the general population. As seronegative partners are likely to benefit from PrEP, future studies should investigate acceptable approaches to PrEP screening in the context of HHC investigation.

## Supporting information

S1 Text Supplementary methods and results. (DOCX)
