# Supplementary material for: Modeled estimates of HIV-serodifferent couples in tuberculosis-affected households in four sub-Saharan African countries
Source: PLOS Glob Public Health. 2024 May 2;4(5):e0002609. doi: 10.1371/journal.pgph.0002609 (PMC11065259; doi:10.1371/journal.pgph.0002609)
Supplement: S1 Text — (DOCX) [file pgph.0002609.s001.docx]

Supplementary material for Zhang, Tseng, et al, “Modeled estimates of HIV-serodifferent couples in tuberculosis-affected households in four sub-Saharan African countries”

Table of Contents

[1. Literature Review 3](#_Toc145674236)

[1.1. Methods 3](#_Toc145674237)

[1.1.1. Search Strategy 3](#_Toc145674238)

[1.1.2. Selection Criteria 3](#_Toc145674239)

[1.2. Results 4](#_Toc145674240)

[Fig A – PRISMA flow diagram for systematic literature review 5](#_Toc145674241)

[1.3 Search summary for viral suppression parameter 5](#_Toc145674242)

[2. Calculating HIV prevalence among people with prevalent TB 6](#_Toc145674243)

[3. *P_SDC_* Calculation 7](#_Toc145674244)

[3.1. $PSDC$ 7](#_Toc145674245)

[Table S1: Data extracted from Demographic and Health Surveys and corresponding calculated values of *P_SDC_* 7](#_Toc145674246)

[3.2 Homogeneity parameter ($\alpha$) 8](#_Toc145674247)

[Table S2: The calculated values of P_SDC_ for Ethiopia, South Africa, Kenya, and Uganda. 9](#_Toc145674248)

[3.3. Proportion of serodifferent couples in TB-affected households 9](#_Toc145674249)

[4. References 10](#_Toc145674250)

# 1. Literature Review

## 1.1. Methods

### 1.1.1. Search Strategy

The PubMed database was searched on October 5, 2021 to identify HIV prevalence estimates for household contacts (HHCs) of tuberculosis (TB) index patients with or without HIV. Four countries were selected for this study due to relatively high HIV burdens and known TB household contact investigation programs: Ethiopia, Kenya, South Africa, and Uganda. The search included peer-reviewed studies published in English since 2010. Studies that were published in non-English were excluded. The key search terms for HIV, tuberculosis, and household contacts used to search PubMed were: (tuberculosis) AND ((HIV) OR (HIV infection*) OR (HIV testing) OR (case*)) AND ((household*) OR (household contact*) OR (home*)) AND ((Ethiopia) OR (Kenya) OR (South Africa) OR (Uganda)) AND (2010:2021[pdat]).

### 1.1.2. Selection Criteria

Search results with titles and abstracts were exported from PubMed and imported into Mendeley Desktop© Version 1.19.8. Title and abstract screening were performed based on the following inclusion criteria: included estimates on prevalence of HIV in HHCs of TB index patients, was a cohort study design, and published in the English language*.* Studies were considered if they included study populations in Ethiopia, Kenya, South Africa, or Uganda. Publications were excluded if they did not include the target countries (Ethiopia, Kenya, South Africa, or Uganda), did not include HIV prevalence estimates in HHCs, did not include TB index patients, contained non-original research (e.g., conference proceedings, clinical guidelines, systematic/literature reviews, editorials, case studies, journal introduction), or were published in a non-English language.

## 1.2. Results

A total of 502 records were retrieved from the database search in PubMed (**Fig A**). All 502 records proceeded to title/abstract screening, from which 19 reports were sought for full-text retrieval. After assessing 19 full texts for eligibility, 9 were excluded. Full-text evaluation resulted in the selection of 10 research studies that met all inclusion criteria [1–10]. Our study population of interest was adult HHCs (defined as individuals who are $\geq$15 years old), but several studies reported HIV prevalence calculated among HHCs of all ages, including children. Upon our request, we received HIV prevalence data stratified by age group ($\geq$15 vs. $<$15 years old) from authors of four studies [5,8–10]. Of the 9 excluded studies, five were excluded because they reported on the same or overlapping cohorts with included studies and four were excluded for lack of age-stratified data. We included one study that used an age cutoff for adults of $\geq$18 years without further adjustment to an age threshold of 15 years [4].

### Fig A – PRISMA flow diagram for systematic literature review


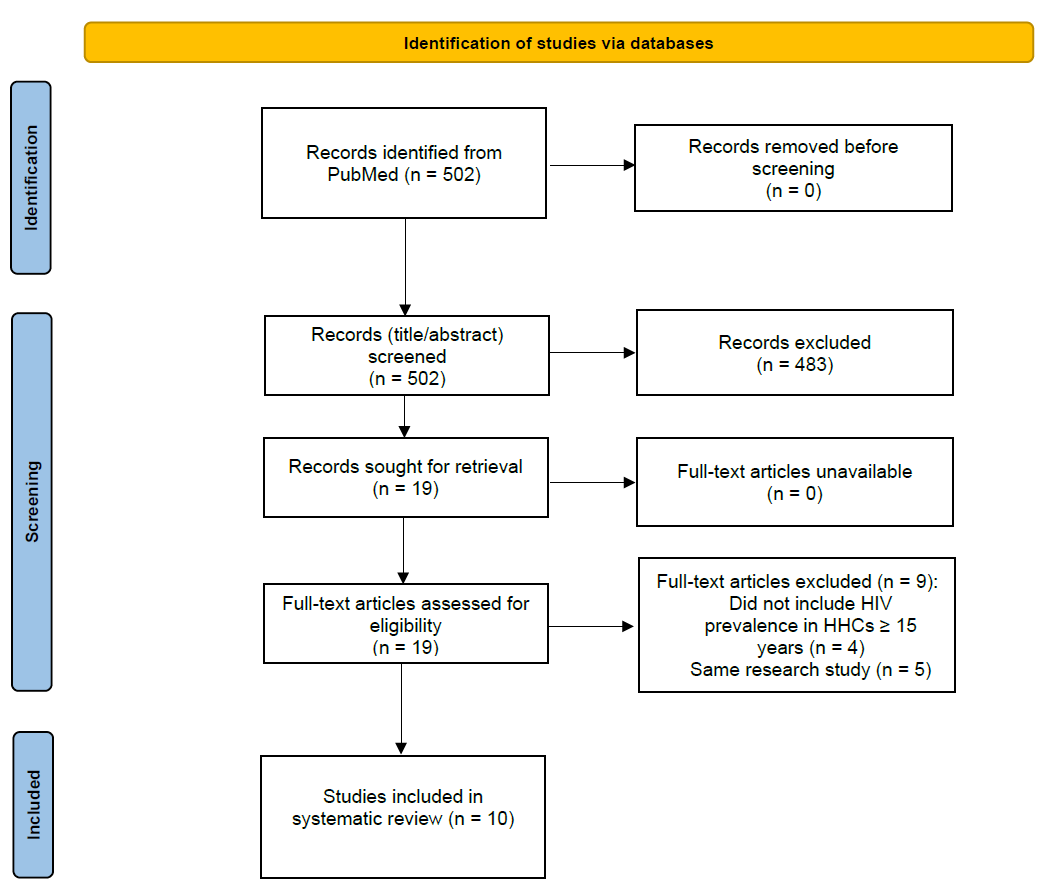


**Fig A.** PRISMA 2020 flow diagram summarizing the selection process for the studies included in this systematic review.

## 1.3 Search summary for viral suppression parameter

We completed a separate search to identify studies reporting the proportion of people living with HIV in TB-affected households without viral suppression. However, this search did not yield studies that could be used to estimate this parameter. Instead, we used a scenario analysis to complete the estimation. The search strategy used in PubMed was:

("Tuberculosis"[Mesh] OR "Antitubercular Agents"[Mesh] OR tuberculosis OR tuberculostatic OR antitubercular OR "anti tubercular") AND HIV AND ("Antiretroviral Therapy, Highly Active"[Mesh] OR "Anti-HIV Agents"[Mesh] OR HAART OR "antiretroviral therap*" OR "anti retroviral therap*" OR "antiretroviral agent*" OR "anti retroviral agent*" OR "anti HIV") AND (discordan* OR serodiscordan* OR (((seropositiv* OR "sero positiv*" OR "HIV positive") AND (seronegativ* OR "sero negativ*" OR "HIV negative")) AND (couple* OR partner* OR spous* OR married OR husband* OR wife OR wives OR union*))).

# 2. Calculating HIV prevalence among people with prevalent TB

HIV prevalence among people with prevalent TB by age and sex was extracted from national TB prevalence survey reports in Kenya and Uganda [11,12]. HIV prevalence among people with prevalent TB was not reported by age and sex in South African national TB prevalence survey [13], so the figure of 28.8% was disaggregated by age and sex in the following steps. First, we assumed the prevalence ratio of HIV among people with prevalent TB vs. HIV prevalence among the general population was constant across each age-sex stratum. Then, HIV prevalence among the general population was extracted from the South African Demographic and Health Survey 2016 [14]. Finally, we applied the HIV prevalence ratio to HIV prevalence among the general population in each age-sex stratum to calculate HIV prevalence among people with prevalent TB by age and sex.

HIV testing was not conducted in the Ethiopia national TB prevalence survey [15]. So, HIV prevalence among people with prevalent TB in Ethiopia was calculated in two steps. First, the HIV prevalence among people with notified TB was extracted from notification data in the Global TB Report [16]. Then, this value was multiplied by the ratio of HIV prevalence among people with prevalent TB versus notified TB from a meta-analysis of national TB prevalence surveys in 7 countries in sub-Saharan Africa [17]. We further disaggregated the HIV prevalence among people with prevalent TB using the same approach as described above for South Africa.

# 3. *P_SDC_* Calculation

## 3.1. $P_{SDC}$

$P_{SDC}$ is the proportion of serodifferent couples (SDCs) among all people living with HIV who are in stable partnerships. Therefore, $P_{SDC}$ is:

$$\frac{number of SDCs}{number of individuals living with HIV who are engaged in stable couples}$$

We calculated $P_{SDC}$ using data for HIV prevalence, prevalence of serodifference, and seroconcordancy data from Demographic Health Survey (DHS) (**Table A**):

$$\frac{Percentage of SDCs}{Percentage of SDCs+2*Percentage of both HIV-positive couples}$$

Therefore, we have 19 $P_{SDC}-P$ pairs, where $P$ is the prevalence of HIV in the 15–49-year-old population.

### Table A: Data extracted from Demographic and Health Surveys and corresponding calculated values of *P_SDC_*

| Country | Year | HIV Prevalence | Prevalence of Serodifference | Prevalence of Seroconcordance | Calculated *P_SDC_* |
| --- | --- | --- | --- | --- | --- |
| Kenya | 2008 | 6.3% | 5.7% | 2.8% | 0.504 |
| Uganda | 2011 | 7.3% | 6.2% | 3.4% | 0.500 |
| Ethiopia | 2016 | 0.9% | 0.8% | 0.3% | 0.570 |
| South Africa | 2016 | 21.2% | 15.7% | 14.6% | 0.350 |
| Lesotho | 2014 | 24.6% | 15.1% | 19.9% | 0.275 |
| Lesotho | 2009 | 23.0% | 16.6% | 18.9% | 0.305 |
| Burkina Faso | 2010 | 1.0% | 1.2% | 0.2% | 0.750 |
| Cameroon | 2018 | 2.7% | 3.3% | 0.8% | 0.673 |
| Cameroon | 2011 | 4.3% | 5.9% | 1.5% | 0.663 |
| Ghana | 2014 | 2.0% | 2.5% | 0.8% | 0.610 |
| Guinea | 2018 | 1.5% | 2.8% | 0.2% | 0.875 |
| Guinea | 2012 | 1.7% | 2.3% | 0.8% | 0.590 |
| Côte d'Ivoire | 2011 | 3.7% | 5.4% | 1.9% | 0.587 |
| Malawi | 2015 | 8.8% | 8.4% | 5.6% | 0.429 |
| Malawi | 2010 | 10.6% | 8.5% | 6.3% | 0.403 |
| Senegal | 2017 | 0.5% | 0.8% | 0.2% | 0.667 |
| Mali | 2012 | 1.1% | 1.7% | 0.2% | 1.000 |
| Tanzania | 2011 | 5.1% | 4.6% | 2.4% | 0.489 |
| Zimbabwe | 2015 | 13.8% | 8.8% | 10.9% | 0.288 |

## 3.2 Homogeneity parameter ($\alpha$)

We used the 19 $P_{SDC}-P$ pairs shown in Table A to model the general relationship between *P_SDC_* and population-level HIV prevalence (P) using the equation:

$$P_{SDC}\left( P \right) \cong\frac{\left( P\left( 1-P \right) \right)^{\alpha}}{P}$$

The homogeneity parameter ($\alpha$) is the only parameter used to model $P_{SDC}$. After we confirmed the $\alpha$, we calculated the corresponding values of $P_{SDC}$for each country using the equation:

$$\frac{\left( P\left( 1-P \right) \right)^{\alpha}}{P}$$

The calculated values of *P_SDC_* for our 4 countries were shown in Table B.

### Table B: The calculated values of P_SDC_ for Ethiopia, South Africa, Kenya, and Uganda.

|  | Ethiopia | South Africa | Kenya | Uganda |
| --- | --- | --- | --- | --- |
| P_SDC_ (Incidence scenario) | 0.028 | 0.317 | 0.194 | 0.153 |
| P_SDC_ (Prevalence scenario) | 0.011 | 0.311 | 0.181 | 0.148 |

## 3.3. Proportion of serodifferent couples in TB-affected households

We additionally calculated the proportion of SDCs in TB-affected households using the following equation:

$$\frac{number of HIV-positive individuals*proportion of adults engaged in stable partnerships * P_{SDC}}{number of all adults *\frac{proportion of adults engaged in stable partnerships}{2}}$$

We accounted for the probability that people aged 15-49 years old engaged in a stable partnership. The probabilities were estimated using the proportion of married/living with partner from DHS reports.

# 4. References

1. Velen K, Lewis JJ, Charalambous S, Page-Shipp L, Popane F, Churchyard GJ, et al. Household HIV testing uptake among contacts of TB patients in South Africa. PLoS One. 2016;11(5).

2. Ochom E, Meyer AJ, Armstrong-Hough M, Kizito S, Ayakaka I, Turimumahoro P, et al. Integrating home HIV counselling and testing into household TB contact investigation: a mixed-methods study. Public Heal Action [Internet]. 2018 Jun 21;8(2):72–8. Available from: https://www.ingentaconnect.com/content/10.5588/pha.18.0014

3. Page-Shipp L, Lewis JJ, Velen K, Senoge S, Zishiri E, Popane F, et al. Household point of care CD4 testing and isoniazid preventive therapy initiation in a household TB contact tracing programme in two districts of South Africa. PLoS One. 2018;13(3).

4. Odera S, Mureithi M, Aballa A, Onyango N, Anzala O, Oyugi J. Latent tuberculosis among household contacts of pulmonary tuberculosis cases in Nairobi, Kenya. Pan Afr Med J. 2020;37.

5. Shapiro AE, Variava E, Rakgokong MH, Moodley N, Luke B, Salimi S, et al. Community-based targeted case finding for tuberculosis and HIV in household contacts of patients with tuberculosis in South Africa. Am J Respir Crit Care Med. 2012;185(10).

6. Thind D, Charalambous S, Tongman A, Churchyard G, Grant AD. An evaluation of “Ribolola”: A household tuberculosis contact tracing programme in North West Province, South Africa. Int J Tuberc Lung Dis. 2012;16(12).

7. Stein CM, Zalwango S, Malone LL, Thiel B, Mupere E, Nsereko M, et al. Resistance and Susceptibility to Mycobacterium tuberculosis Infection and Disease in Tuberculosis Households in Kampala, Uganda. Am J Epidemiol [Internet]. 2018 Jul 1;187(7):1477–89. Available from: https://academic.oup.com/aje/article/187/7/1477/4786120

8. MacPherson P, Lebina L, Motsomi K, Bosch Z, Milovanovic M, Ratsela A, et al. Prevalence and risk factors for latent tuberculosis infection among household contacts of index cases in two South African provinces: Analysis of baseline data from a cluster-randomised trial. PLoS One. 2020;15(3).

9. Opollo VS, Wu X, Hughes MD, Swindells S, Gupta A, Hesseling A, et al. HIV testing uptake among the household contacts of multidrug-resistant tuberculosis index cases in eight countries. Int J Tuberc lung Dis Off J Int Union against Tuberc Lung Dis. 2018 Dec;22(12):1443–9.

10. Warria K, Nyamthimba P, Chweya A, Agaya J, Achola M, Reichler M, et al. Tuberculosis disease and infection among household contacts of bacteriologically confirmed and non-confirmed tuberculosis patients. Trop Med Int Heal. 2020;25(6).

11. Enos M, Sitienei J, Ong’ang’o J, Mungai B, Kamene M, Wambugu J, et al. Kenya tuberculosis prevalence survey 2016: Challenges and opportunities of ending TB in Kenya. PLoS One [Internet]. 2018;13(12):e0209098. Available from: http://www.ncbi.nlm.nih.gov/pubmed/30586448

12. Uganda Ministry of Health. The Uganda National Tuberculosis Prevalence Survey, 2014-2015 survey report. 2015; Available from: http://library.health.go.ug/publications/tuberculosis/uganda-national-tuberculosis-prevalence-survey-2014-2015-survey-report

13. Moyo S, Ismail F, Van der Walt M, Ismail N, Mkhondo N, Dlamini S, et al. Prevalence of bacteriologically confirmed pulmonary tuberculosis in South Africa, 2017-19: a multistage, cluster-based, cross-sectional survey. Lancet Infect Dis [Internet]. 2022 May 17; Available from: http://www.ncbi.nlm.nih.gov/pubmed/35594897

14. South Africa National Department of Health. South Africa Demographic and Health Survey 2016 [Internet]. 2019. Available from: https://dhsprogram.com/pubs/pdf/FR337/FR337.pdf

15. Kebede AH, Alebachew Z, Tsegaye F, Lemma E, Abebe A, Agonafir M, et al. The first population-based national tuberculosis prevalence survey in Ethiopia, 2010-2011. Int J Tuberc Lung Dis [Internet]. 2014 Jun;18(6):635–9. Available from: http://www.ncbi.nlm.nih.gov/pubmed/24903931

16. World Health Organization. Global tuberculosis report 2021. [Internet]. Geneva; 2021. Available from: https://www.who.int/teams/global-tuberculosis-programme/tb-reports

17. Law I, Floyd K, Abukaraig EAB, Addo KK, Adetifa I, Alebachew Z, et al. National tuberculosis prevalence surveys in Africa, 2008–2016: an overview of results and lessons learned. Trop Med Int Heal [Internet]. 2020 Nov 12;25(11):1308–27. Available from: https://onlinelibrary.wiley.com/doi/10.1111/tmi.13485
